# Multi-Group Gorilla Troops Optimizer with Multi-Strategies for 3D Node Localization of Wireless Sensor Networks

**DOI:** 10.3390/s22114275

**Published:** 2022-06-03

**Authors:** Qingwei Liang, Shu-Chuan Chu, Qingyong Yang, Anhui Liang, Jeng-Shyang Pan

**Affiliations:** 1 College of Computer Science and Engineering, Shandong University of Science and Technology, Qingdao 266590, China; liangqw@sdust.edu.cn (Q.L.); scchu0803@sdust.edu.cn (S.-C.C.); yang_qy@sdust.edu.cn (Q.Y.); 2 College of Electronic and Information Engineering, Shandong University of Science and Technology, Qingdao 266590, China; liangah8@sdust.edu.cn; 3 Department of Information Management, Chaoyang University of Technology, Taichung 413310, Taiwan; 4 Fujian Provincial Key Laboratory of Big Data Mining and Applications, Fujian University of Technology, Fuzhou 350118, China

**Keywords:** artificial gorilla troops optimizer, opposition-based learning, parallel communication strategy, wireless sensor networks, 3D node localization

## Abstract

The localization problem of nodes in wireless sensor networks is often the focus of many researches. This paper proposes an opposition-based learning and parallel strategies Artificial Gorilla Troop Optimizer (OPGTO) for reducing the localization error. Opposition-based learning can expand the exploration space of the algorithm and significantly improve the global exploration ability of the algorithm. The parallel strategy divides the population into multiple groups for exploration, which effectively increases the diversity of the population. Based on this parallel strategy, we design communication strategies between groups for different types of optimization problems. To verify the optimized effect of the proposed OPGTO algorithm, it is tested on the CEC2013 benchmark function set and compared with Particle Swarm Optimization (PSO), Sine Cosine Algorithm (SCA), Whale Optimization Algorithm (WOA) and Artificial Gorilla Troops Optimizer (GTO). Experimental studies show that OPGTO has good optimization ability, especially on complex multimodal functions and combinatorial functions. Finally, we apply OPGTO algorithm to 3D localization of wireless sensor networks in the real terrain. Experimental results proved that OPGTO can effectively reduce the localization error based on Time Difference of Arrival (TDOA).

## 1. Introduction

Solving optimization problems by simulating the behavior of biological groups has become a new research hotspot, and a theoretical system with swarm intelligence as the core has been formed. Observations and studies of biological groups have found that swarm intelligence generated by complex behaviors such as cooperation and competition among individuals in biological groups can often provide efficient solutions to some specific problems. Research on swarm intelligence has made breakthroughs in some practical application fields, and more excellent optimization algorithms [1] have been proposed and applied in various fields such as wireless sensor networks, robotics, and electric power. For example, Particle Swarm Optimization (PSO) [2,3,4], Whale Optimization Algorithm (WOA) [5,6,7], Sine Cosine Algorithm (SCA) [8,9,10], Flower Pollination Algorithm (FPA) [11,12], Differential Evolution Algorithm (DE) [13,14,15,16], Cat Swarm Optimization (CSO) [17,18,19,20], Bat Algorithm (BA) [21,22,23], Artificial Bee Colony (ABC) [24,25], Pigeon-Inspired Optimization (PIO) [26,27], and Ant Colony Optimization (ACO) [28,29].

So far many optimization algorithms have been proposed and achieved excellent results. So why do we still need to research and come up with new optimization algorithms? According to the No Free lunch (NFL) theorem proposed by Wolpert and Macready [30,31], there is no optimization algorithm that can solve a wide variety of problems. In 2021, Benyamin Abdollahzadeh and Farhad Soleimanian Gharehchopogh proposed a new optimization algorithm, which is called Artificial Gorilla Troops Optimizer (GTO) [32]. The new optimization algorithm is inspired by the social intelligence of gorilla troops in nature. The algorithm uses mathematical formulas to represent the collective life of gorillas and designs new mechanisms to exploration and exploitation. The experimental results show that the convergence speed and optimal solution of GTO significantly outperform some known optimization algorithms.

Although GTO has achieved excellent results in many research fields, many experiments also show that GTO is easy to obtain local optimal solutions when solving complex problems. Hamid R. Tizhoosh from the University of Waterloo proposed the opposition-based learning [33] strategy in 2005, which is used to generate the reverse solution of the particle, and construct the reverse swarm corresponding to the current swarm. This strategy helps to search for more effective areas to improve the diversity of groups and enhance the global exploration ability of the algorithm. Technologies related to evolutionary computing emerge in an endless stream, the parallel strategy has become a particularly important method to improve intelligent optimization algorithm. The parallel strategy means that the population is divided into multiple groups, and each group is iteratively updated independently. The significant advantage of this strategy is not only to strengthen the diversity of particle swarms, but also greatly enhances the global search ability of the algorithm. Especially when it comes to more complicated optimization problems, the parallel strategy is a particularly effective method to further upgrade the efficiency of the algorithm and find the optimal solution. Many studies prove that the parallel strategy has been successfully applied to a variety of optimization algorithms, such as parallel PSO [34] and parallel CSO [35]. Inspired by these strategies, we introduces opposition-based learning and parallel mechanism to enhance the performance of GTO, which is called OPGTO. OPGTO is obviously superior to GTO in terms of convergence speed and accuracy of solving the optimal solution, and has obvious advantages over some well-known optimization algorithms.

With the rapid development and maturity of communication technology, embedded computing technology and sensor technology, micro-sensors with sensing, computing and communication capabilities have begun to appear around the world. Wireless sensor networks composed of these tiny sensors have attracted great attention. WSN integrates sensor technology, embedded computing technology, distributed information processing technology and communication technology. It can collaboratively monitor, perceive and collect information of various environments or monitoring objects in the network distribution area in real time. Therefore, this network system can be widely used in various fields, such as forest fire prevention, ocean detection and predict nature disaster [36], etc.

In many scenarios, sensor nodes are randomly deployed in a certain area. Since nodes cannot know their location in advance, they need to obtain their location information through localization technology after deployment. It is known to all that the most common localization technology is GPS, which can locate nodes through satellites and achieve relatively high accuracy. However, due to the high energy consumption and cost of GPS receivers, it is impossible to equip each node with a GPS receiver. Therefore, WSN usually consist of anchor nodes with GPS and unknown nodes without GPS. Through some localization technologies, unknown nodes can determine their position based on anchor nodes. The location technology of WSN consists of ranging phase and evaluation phase. In the ranging phase, multiple sending nodes can transmit signals at the same time, and the receiving nodes can calculate the distance based on the time difference between the arrival of the signals and their propagation speed, which is called Time Difference of Arrival (TDOA) [37,38]. This paper applies OPGTO to solve the 3D localization problem in WSN and can achieve lower localization errors through the idea of TDOA.

The remainder of the paper is organized as follows. In Section 2, we will elaborate on the GTO algorithm in detail. Section 3 elaborates on the opposition-based learning and parallel mechanism in OPGTO, and how the new algorithm can be combined with TDOA and applied to 3D localization. Section 4 discusses in detail the simulation results of OPGTO on the CEC2013 benchmark functions and how to reduce the error of 3D localization in WSN. Section 5 discusses the innovations of our approach. Section 6 mainly summarizes and looks forward to this paper.

## 2. Related Work

In this section, will first introduce the design ideas and implementation steps of the GTO algorithm. Then, we will analyze how to use TDOA for node localization in WSN.

### 2.1. Artificial Gorilla Troops Optimizer

The GTO algorithm includes the exploration phase and the exploitation phase, Equations (1)–(13) [32] describe the main idea of the algorithm. The exploration phase is mainly used to perform a global search of the space. It uses three different mechanisms, includes migrating to an unknown position, migrating to a known position, and moving to the position of other gorillas. Equation (1) simulates the exploitation phase.

(1) $$GX\left(t+1\right)=\left\{\begin{array}{cc} \left(ub-lb\right)\times r_{1}+lb, & r<p,\\ \left(r_{2}-C\right)\times X_{r}\left(t\right)+L\times H, & r\geq 0.5,\\ X\left(i\right)-L\times \left(L\times \left(X\left(t\right)-GX_{r}\left(t\right)\right)+r_{3}\times (X\left(t\right)-GX_{r}\left(t\right))\right), & r<0.5. \end{array}\right.$$

In Equation (1), *X(t)* represents the gorilla current position and GX(t+1) is the gorilla position in the t+1 iteration. *p* a parameter between 0 and 1 that determines which migration mechanism to choose. *lb* and *ub* are the lower and upper bounds of the variable, respectively. *X${}_{r}$* is a randomly selected gorilla member from the population and *GX_r_* is the randomly selected gorilla candidate position vector. *r*_1_, *r*_2_, *r*_3_, and *r* are random values in the range 0 to 1 updated on each iteration. Moreover, *C*, *L*, and *H* are calculated from the following equations.

(2) $$C=F\times \left(1-\frac{It}{MaxIt}\right),$$

(3) $$F=cos(2\times r_{4})+1,$$

(4) L=C×l,

(5) H=Z×X(t),

(6) Z=[−C,C].

In Equation (2), *It* is the current number of iterations and *MaxIt* is the total number of iterations of the algorithm. In Equation (3) and (4), *r${}_{4}$* and *l* are random values between 0 to 1 updated on each iteration. In Equation (6), *Z* is a random value in the range −C to *C*. At the end of the exploration phase, the algorithm calculated the fitness value of all *GX* solutions, and if the fitness value is GX(t)<X(t), the *X(t)* solution is replaced by the *GX(t)* solution.

The exploitation process of the GTO algorithm uses two mechanisms, following silverback gorilla and competing for adult female gorillas. The mechanism is selected by comparing the *C* value calculated by the Equation (2) with the parameter *W* set in advance. If C≥W, the GTO algorithm uses the following silverback gorilla mechanism, but if C<W, competing for adult female gorillas is selected. Equation (7) is used to simulate Follow the silverback gorilla.

(7) $$GX\left(t+1\right)=L\times M\times (X\left(t\right)-X_{silverback})+X\left(t\right),$$

(8) $$M={\left({\left|\frac{1}{N}\sum_{i=1}^{N}GX_{i}\left(t\right)\right|}^{g}\right)}^{\frac{1}{g}},$$

(9) $$g=2^{L}.$$

In Equation (7), $X_{silverback}$ is the silverback gorilla position. In Equation (8), $GX_{i}\left(t\right)$ is the position of each candidate gorilla in iteration *t* and *N* is the total number of gorillas. Furthermore, Equation (10) is used to simulate Compete with adult female gorillas.

(10) $$GX\left(i\right)=X_{silverback}-\left(X_{silverback}\times Q-X\left(t\right)\times Q\right)\times A,$$

(11) $$Q=2\times r_{5}-1,$$

(12) A=β×E,

(13) $$E=\left\{\begin{array}{cc} N_{1}, & r\geq 0.5,\\ N_{2}, & r<0.5. \end{array}\right.$$

In Equation (11), *r${}_{5}$* is a random value between 0 to 1 updated on each iteration. In Equation (12), β is a parameter to be given value. In Equation (13), if rand≥0.5, *E* is a random value in the normal distribution and the dimensions of the problem, but if rand<0.5, *E* is a random value chosen from a normal distribution. At the end of the exploitation phase, the algorithm calculated the fitness value of all *GX* solutions. If the fitness value is GX(t)<X(t), the *X(t)* solution is replaced by the *GX(t)* solution and the optimal solution selected in the entire population is regarded as the silverback gorilla.

### 2.2. 3D Localization of Wireless Sensor Network

**Figure 1:**
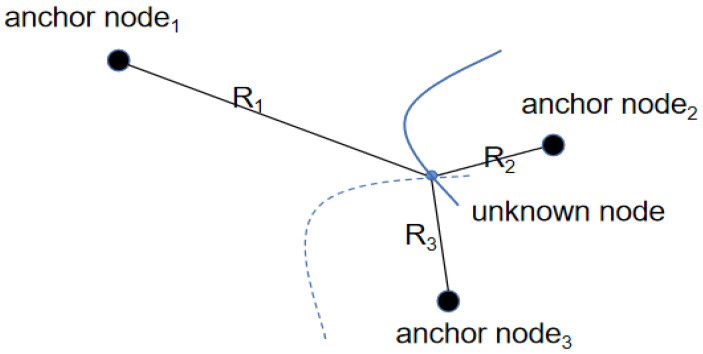
The principle of TDOA.

In this paper, OPGTO is applied to the 3D node localization in WSN. The new algorithm uses the TDOA method to locate the nodes. The TDOA localization method is to determine the location of the unknown node by measuring the propagation time difference of the signal from the unknown node to the two anchor nodes. According to the distance difference corresponding to this propagation time difference, a hyperbola with the corresponding two anchor nodes as the focus can be obtained. Therefore, if we want to get the estimated position value of the unknown node, we only need to measure the difference between the two sets of TDOA. Two hyperbolas can be obtained from these two sets of TDOA data values, and the intersection of these two hyperbolas is the estimated position of the node to be measured. The schematic diagram of this method is shown in Figure 1.

## 3. Artificial Gorilla Troops Optimizer with Opposition-Based Learning and Parallel Strategy (OPGTO) for 3D Localization

In this section, we propose the opposition-based learning strategy for expanding the search space of the algorithm. On this basis, we propose two communication strategies based on the idea of parallel to increase the diversity of the population and the exploration ability of the algorithm. Finally, we apply the new algorithm to 3D localization.

### 3.1. Opposition-Based Learning

In many cases, population generates individuals in a random fashion. However, if the algorithm starts its search from a location far from the optimal solution, especially in the worst case where the initial solution is located opposite the optimal solution, the search will take a considerable amount of time. Therefore, the best strategy is to search in all directions, or more specifically, in the opposite direction. Following this idea, this paper uses opposition-based learning in the two stages of population initialization and algorithm stagnation. Opposition-based learning mainly completes the following three parts.

- Initial position of individual gorillas is $X_{i,j}$, i=1,2,⋯,N, j=1,2,⋯,D. *N* is the population size, and *D* is the dimension of the search space. The population at this time is called $P_{1}$.
- The population is generated according to Equation (14) and called $P_{2}$.
  (14) $$X_{i,j}^{{}^{\prime }}=lb\left(j\right)+ub\left(j\right)-rand\times X_{i,j},$$
  On the basis of Hamid R. Tizhoosh’s method [33], we used *rand* to broaden the search space. In Equation (14), *lb(j)* is the lower bound of the *j*th dimension and *ub(j)* is the upper bound of the *j*th dimension. *rand* is a random value in the range 0 to 1. *X${}_{i,j}^{{}^{\prime }}$* is the opposite gorilla.
- In the population *P${}_{1}$* and *P${}_{2}$*, the algorithm selects the number *N* of individuals with the best fitness to form an initialization population.

In the population initialization phase, the algorithm first generates an initial population, and then performs opposition-based learning.

According to the flow of the algorithm, OPGTO will gradually approach the initial solution to the optimal solution. However, due to the limitation of various factors, the algorithm is prone to premature phenomenon and falls into the local optimal solution. This leads to the evolutionary stagnation. Therefore, when the algorithm stagnates, OPGTO will perform opposition-based learning to expand the scope of exploration and escape the current local optimum solution as much as possible.

**Figure 2:**
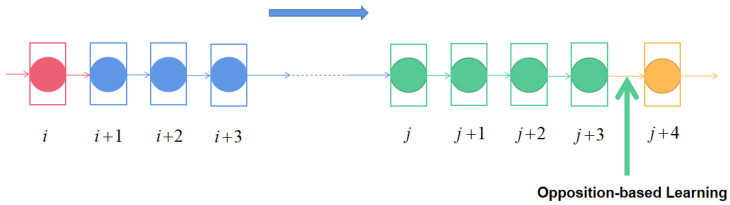
Opposition-based learning with sliding window strategy.

First, OPGTO needs to perform stagnant detection. In order to design a method to effectively detect the stagnation of the algorithm, this paper introduces the sliding window strategy. The size of the window is set to 4. When the optimal values calculated by the algorithm are all equal in the adjacent 4 iterations, it means that the algorithm is stagnant. At this time, the original population is updated by opposition-based learning. Figure 2 describes the specific process.

### 3.2. Parallel Strategy

This section mainly introduces the parallel strategy of OPGTO. In this paper, the parallel strategy is mainly expressed as a variety of intergroup communication strategies, including multi-group merge communication strategy and multi-group competition communication strategy. The communication strategies can effectively update information between each group, so using these can improve the performance of the algorithm.

#### 3.2.1. Multi-Group Merge Communication Strategy

Some communication methods in parallel strategies generally attempt to change the way information is exchanged between groups rather than changing the number of groups. Intelligent optimization algorithms are usually inspired by the evolutionary process of biological populations. In the real development process, there are often mergers and fusions between populations, which also leads to the continuous development of populations. In the process, although weaker populations are swallowed up, they join stronger groups that are better adapted to the development of nature.

**Figure 3:**
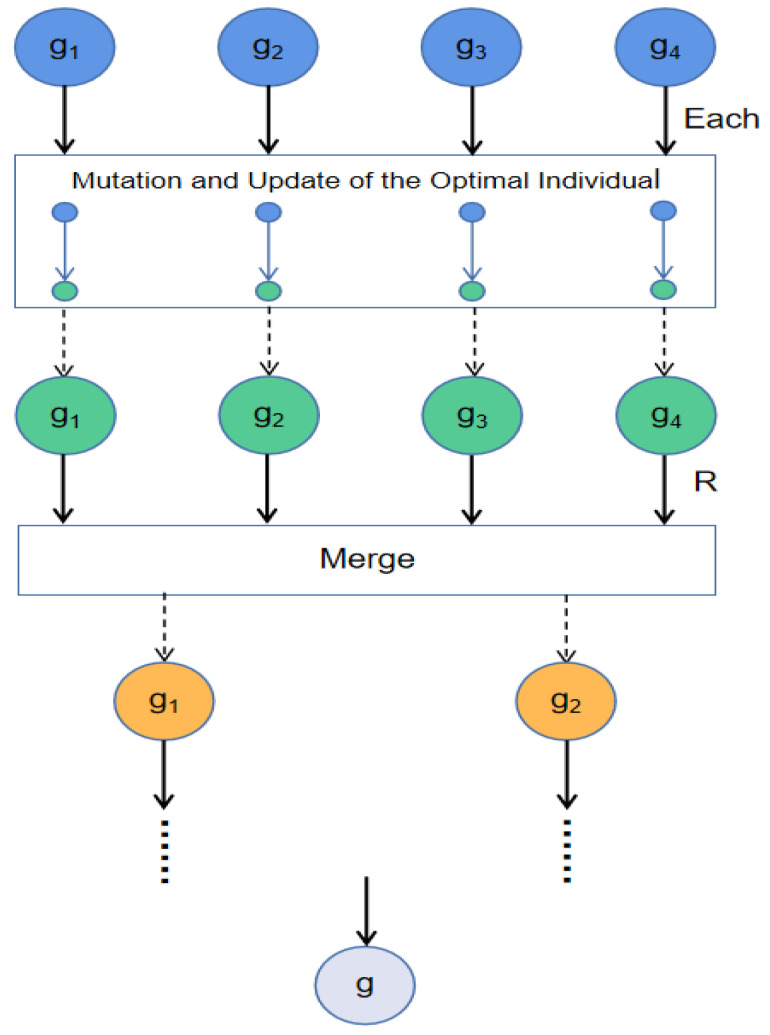
The running process of the population merge communication strategy.

Inspired by this, the communication strategy divides the initial population into some groups. Firstly, Each group explores independently and exploits its own optimal individual. After a certain degree of evolution, every two neighbor groups complete the communication strategy and merge into a new group. For example, the first group and the second group will be merged into the new first group after the communication is completed. With this idea, the new group not only retains the effective information of the two groups after merging, but also can conduct new explorations. At the same time, the new group needs to update the global best individual, which is selected from all previous groups. Figure 3 helps us better understand the merge process.

In Figure 3, each group is individually updated iteratively, and when the number of iterations is multiple of *R*, all groups perform merge operations. In addition, the algorithm performs mutation and update operations after each iteration. We set the value of R in Section 4.1. Mutation refers to selecting the optimal individuals from each group, and then subjecting them to Gaussian mutation and Cauchy mutation to expand the searchable range of optimal values. Finally, the individual with the best fitness is selected from the original optimal individual and the mutant individual as the optimal individual of each group. Li C [39] used Cauchy and Gaussian mutation to update the particles, and we’ve made some improvements based on that. Equations (15)–(17) describe the mutation operation.

(15) $$MX_{best}^{t}=X_{best}^{t}+X_{best}^{t}\times \left(\lambda_{1}Cauchy\left(0,1\right)+\lambda_{2}Gauss\left(0,1\right)\right),$$

(16) $$\lambda_{1}=\frac{T-t}{T},$$

(17) $$\lambda_{2}=\frac{t}{T}.$$

In Equation (15), *X${}_{best}^{t}$* is the original optimal individual and *MX${}_{best}^{t}$* represents the mutant individual, Cauchy(0,1) is a random value from the standard Cauchy distribution and Gauss(0,1) is a random value from the standard Gaussian distribution. In addition, $\lambda_{1}$ and $\lambda_{2}$ are calculated from Equations (16) and (17). Among them, *T* is the maximum number of iterations and *t* is the current number of iterations. In the iterative process of the algorithm, $\lambda_{1}$ gradually decreases as the number of iterations increases, but $\lambda_{2}$ gradually increases. This approach can make the algorithm escape the current stagnation position and enhance the ability of global exploration.

#### 3.2.2. Multi-Group Competition Communication Strategy

**Figure 4:**
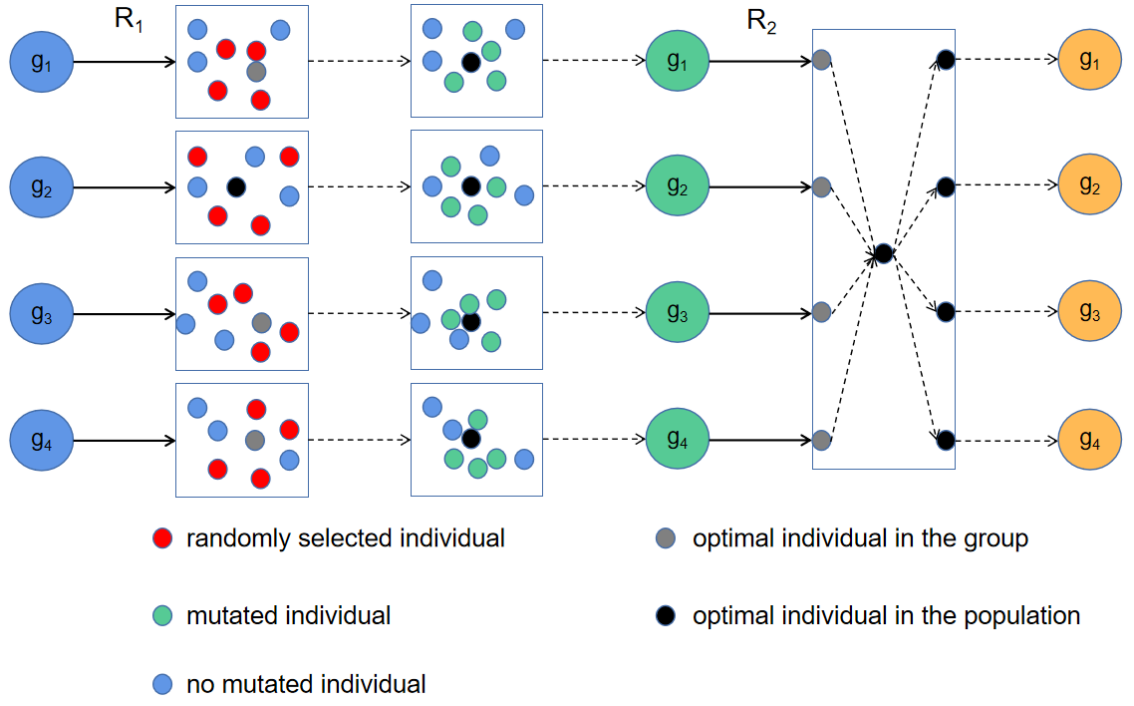
The running process of the population competition communication strategy.

In the process of biological development and evolution, competition among populations is an important way to promote individuals to reform of nature. Nature uses competition to select individuals, which means that individuals with good fitness will have more resources, while individuals with poor fitness will be gradually eliminated. In addition, the genes of individuals with good fitness will be spread, because more and more individuals will learn their behavior and evolve gradually. Therefore, we designs a communication strategy that simulates this phenomenon and Figure 4 shows the detailed idea of it.

In this strategy, the entire population is firstly divided into multiple groups and the iterations of the GTO algorithm are performed separately. The update method of PWOA [40] gave us a lot of inspiration. When the number of iterations is a multiple of *R${}_{1}$*, the following operations are performed:

(1) Select the optimal individual in each group and compare them to get the optimal individual in the entire population.
(2) Randomly select some individuals in each group and mutate them according to Equation (18):
  (18) $$X_{i,d}^{t+1}={X_{best}}_{d}^{t}\times \left(0.2\cdot rand+0.8\right).$$
  where the *X${}_{i,d}^{t+1}$* indicates the value of a random individual in the *d*th dimension at the (t+1)th iteration, *i* is a random number between 0 and the group size. The *X${}_{best}$${}_{d}^{t}$* represents the value of the optimal individual of the population in the *d*th dimension at the *t*th iteration and rand∈[0,1]. This propagation strategy is to randomly select the same number of individuals from each group and learn the optimal individual in the population in different dimensions. In Figure 3, the mutated individual will be closer to the optimal individual in the whole population than before.
(3) Each group’s the optimal individual is updated according to the following equation. *X*(*g*)${}_{best}^{t+1}$ indicates the optimal individual in the *g*th group at the (*t*+1)th iteration and *X*${}_{best}^{t}$ is the optimal individual in the population at the *t*th iteration.
  (19) $$X{\left(g\right)}_{best}^{t+1}=X_{best}^{t}.$$

Then, when the number of iterations of is a multiple of *R${}_{2}$*, *X${}_{best}^{t+1}$* and *X(g)${}_{best}^{t+1}$* are updated by the following equation. In Equation (20), *fitness(X)* means calculating the value of *X* in the fitness function.

(20) $$X_{best}^{t+1}=\left\{\begin{array}{cc} X{\left(g\right)}_{best}^{t+1}, & fitness\left(X{\left(g\right)}_{best}^{t+1}\right)<fitness\left(X_{best}^{t}\right),\\ {X_{best}}^{t}, & fitness\left(X{\left(g\right)}_{best}^{t+1}\right)\geq fitness(X_{best}^{t}). \end{array}\right.$$

(21) $$X{\left(g\right)}_{best}^{t+1}=X_{best}^{t+1}.$$

### 3.3. Apply the OPGTO in 3D Localization

In this paper, OPGTO is used to reduce TDOA-based localization errors in WSN. Inspired by PCSCA [9], the error values is calculated from Equations (22)–(24). In Equations (22) and (23), *R${}_{i}$* represents the distance from the unknown node to the anchor node *i* that it can visit, and *D${}_{i}$* represents the distance from the algorithm estimated node to the anchor node *i* that the unknown node can visit. Since TDOA uses the method of signal arrival time difference for positioning, the signal will inevitably be interfered during the transmission process. Therefore, we set random numbers *Noise${}_{1}$* and *Noise${}_{2}$* to simulate the disturbance of the signal. In order to facilitate the calculation, we take the first anchor node that the unknown node can visit as the benchmark, and use other anchor nodes that the unknown node can visit to calculate the difference with this benchmark. *error${}_{1,i}$* represents the error between the unknown node and node *i*, and *error${}_{2,i}$* represents the error between the node estimated by the algorithm and node *i*.

(22) $$error_{1,i}=R_{i+1}-R_{1}+Noise_{1},$$

(23) $$error_{2,i}=D_{i+1}-D_{1}+Noise_{2},$$

(24) $$error=\sum_{i=1}^{N-1}\left(\sqrt{{\left(error_{1,i}-error_{2,i}\right)}^{2}}\right).$$

*R${}_{i}$* and *D*${}_{i}$ are calculated according to Equations (25) and (26). $\left(x_{i},y_{i},z_{i}\right)$ is the location of anchor node *i*. $\left(X_{1},Y_{1},Z_{1}\right)$ represents the location of the unknown node, and $\left(X_{2},Y_{2},Z_{2}\right)$ represents the location of the node estimated by the algorithm.

(25) $$R_{i}=\sqrt{{\left(X_{1}-x_{i}\right)}^{2}+{\left(Y_{1}-y_{i}\right)}^{2}+{\left(Z_{1}-z_{i}\right)}^{2}},$$

(26) $$D_{i}=\sqrt{{\left(X_{2}-x_{i}\right)}^{2}+{\left(Y_{2}-y_{i}\right)}^{2}+{\left(Z_{2}-z_{i}\right)}^{2}},$$

Positioning in 3D space is often more complex than in 2D space, so the first thing we have to consider is the obstacle problem. For example, in disaster rescue, the signal may be interfered by obstacles when a node in the wireless sensor network communicates with other nodes, which will cause the node to fail to connect with other nodes normally. In other words, the node cannot send its own location information, so that the localization cannot be completed. Therefore, it is very important to check whether there are obstacles between nodes. For obstacle judgment, people often use the line-of-sight (LOS) [41] algorithm for computation.

**Figure 5:**
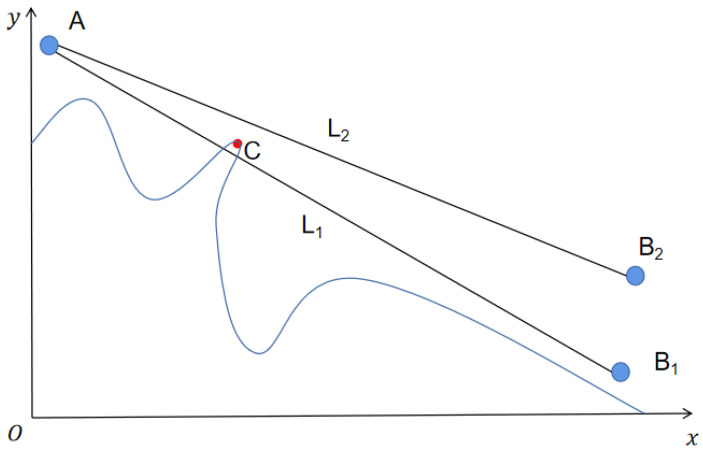
Judging obstacles based on LOS.

The principle of the LOS method is shown in Figure 5. There are two nodes *A* and *B* that need to communicate. If a message is sent from node *A* to node *B*, the most intuitive way is to treat the two nodes as coordinate points and draw a straight line *L* to connect point *A* and point *B*. If any terrain point *C* between two points is above the straight line *L*, then terrain point *C* is a communication obstacle between point *A* and point *B*. Conversely, if there is no terrain point *C* is above the straight line *L*, then we can consider that there is no obstacle between point *A* and point *B*. In Figure 5, the communication between node *A* and node *B${}_{1}$* is affected because there is an obstacle *C*, but node *A* and node *B${}_{2}$* can communicate normally.

## 4. Results

This section shows the simulation results of OPGTO under the CEC2013 [42] test function set and the practical application effect of TDOA in 3D localization.

### 4.1. Results of Benchmark Functions

This paper uses 28 classical mathematical test functions from CEC2013 to verify the performance of the new algorithm. CEC2013 has a total of 28 test functions. F1–F5 are ordinary unimodal functions. F6–F20 are some basic multimodal functions that can effectively detect whether the algorithm can escape from the local optimal solution. Moreover, F21–F28 are some complex functions, which can effectively test the global search ability of the algorithm. All experiments have been tested and executed using the Matlab R2018a, laptop computer running Windows 10 64-bit with an Intel Core i7-8750H 2.20 GHz processor and 8.00 GB RAM.

To evaluate the performance of the new algorithm in detail, we compare OPGTO with the traditional optimization algorithms PSO, WOA, SCA and the original GTO algorithm. Table 1 describes the initial parameters [32,40] for these comparison algorithms. In order to ensure the fairness of the test, each algorithm is independently run 20 times under the same hardware environment to obtain the average value, which represents the performance of the algorithm.

The OPGTO algorithm proposed in this paper uses opposition-based learning for expanding the search range and two communication strategies for communication. The first communication strategy of OPGTO is multi-group merge communication strategy, which reduces the number of groups from 4 to 1. Since the number of iterations is 2000, set *R* to 700. The second communication strategy of OPGTO is multi-group competition communication strategy (OPGTOS2), which requires multiple individuals to gradually approach the optimal individual. So *R${}_{1}$* is set to 100 and *R${}_{2}$* is set to 250. In this way, inter-group communication is more frequent and the algorithm is more likely to escape from local optimal solutions. Algorithm 1 introduces the execution process of OPGTOS1 and Algorithm 2 introduces the execution process of OPGTOS2.
**Algorithm 1** OPGTOS1**Input:** The maximum number of iterations *T*, the population size *N*, the lower boundary *lb*, the upper boundary *ub*, the dimension *dim*, the objective function *fobj*;1: Initialization: Generate the individual $X_{i}\left(i=1,2,\cdots ,N\right)$.2: %Opposition-based learning3: Calculate the opposition-based individual $X_{i}^{{}^{\prime }}\left(i=1,2,\cdots ,N\right)$ using Equation (14). Select *N* individuals with the best fitness from *X${}_{i}$* and *X${}_{i}^{{}^{\prime }}$*;4: Divide the population into *g* groups, every group is Group(j)(j=1,2,⋯,g);5: The best gorilla in each group is *Group(j).silverback* and its fitness value is *Group(j).bestfit*;6: t = 1;7: **while** t < T **do**8:     %Multi-group merge communication strategy9:     **if** t == R **then**10:         j = 011:         **for** i = 1:2:g **do**12:            j = j + 113:            Merge groups14:            %Update the optimal individual for each group15:            *Group(j).silverback* = *BestGorilla*16:            *Group(j).bestfit* = *BestFitness*17:         **end for**18:         g = g/219:     **end if**20:     **for** i = 1:g **do**21:         Update *Group(i)* using GTO22:         Mutate *Group(i).silverback${}^{{}^{\prime }}$* using Equation (15)23:         Select the best fit individual from *Group(i).silverback${}^{{}^{\prime }}$* and *Group(i).silverback*24:         %Update the optimal individual for the entire population25:         **if** *Group(i).BestFitness*<*BestGorilla* **then**26:            *BestGorilla*=*Group(i).silverback*27:            *BestFitness*=*Group(i).bestfit*28:         **end if**29:         **if** t > 100 **then**30:            Opposition-based learning with sliding window strategy31:         **end if**32:     **end for**33: **end while****Output:***BestGorilla* and *BestFitness*.
**Algorithm 2:** OPGTOS2**Input:** The maximum number of iterations *T*, the population size *N*, the lower boundary *lb*, the upper boundary *ub*, the dimension *dim*, the objective function *fobj*;1: Initialization: Generate the individual $X_{i}\left(i=1,2,\cdots ,N\right)$.2: %Opposition-based learning3: Calculate the opposition-based individual $X_{i}^{{}^{\prime }}\left(i=1,2,\cdots ,N\right)$ using Equation (14). Select *N* individuals with the best fitness from *X${}_{i}$* and *X${}_{i}^{{}^{\prime }}$*;4: Divide the population into *g* groups, every group is Group(j)(j=1,2,⋯,g);5: The best gorilla in each group is *Group(j).silverback* and its fitness value is *Group(j).bestfit*;6: t = 1;7: **while** t < T **do**8:     %Multi-group competition communication strategy9:     **for** i = 1:g **do**10:         **if** t == R${}_{1}$ **then**11:            Randomly select some individuals in *Group(i)*12:            Mutate the selected individuals using Equation (18)13:            Update *Group(i).silverback* using Equation (19)14:         **end if**15:         **if** t == R${}_{2}$ **then**16:            Update *BestGorilla* using Equation (20)17:            Update *Group(i).silverback* using Equation (21)18:         **end if**19:     **end for**20:     **for** i = 1:g **do**21:         Update *Group(i)* using GTO22:         %Update the optimal individual for the entire population23:         **if** *Group(i).BestFitness*<*BestGorilla* **then**24:            *BestGorilla*=*Group(i).silverback*25:            *BestFitness*=*Group(i).bestfit*26:         **end if**27:         **if** t > 100 **then**28:            Opposition-based learning with sliding window strategy29:         **end if**30:     **end for**31: **end while****Output:***BestGorilla* and *BestFitness*.

In the experiment, in order to test the optimization ability of the new algorithm in different dimensions, the new algorithm and the comparison algorithm were tested in 10, 30 and 50 dimensions respectively. In Table 2, all algorithms are tested in 10 dimensions. Compared to other algorithms, OPGTOS1 achieves 16 better solutions and 2 identical solutions, while OPGTOS2 achieves 7 better solutions and 2 identical solutions. Compared with the GTO algorithm, OPGTOS1 achieved 24 better solutions and 2 identical solutions, and OPGTOS2 achieved 22 better solutions and 2 identical solutions, which shows that the improved strategy proposed in this paper is effective.

**Figure 6:**
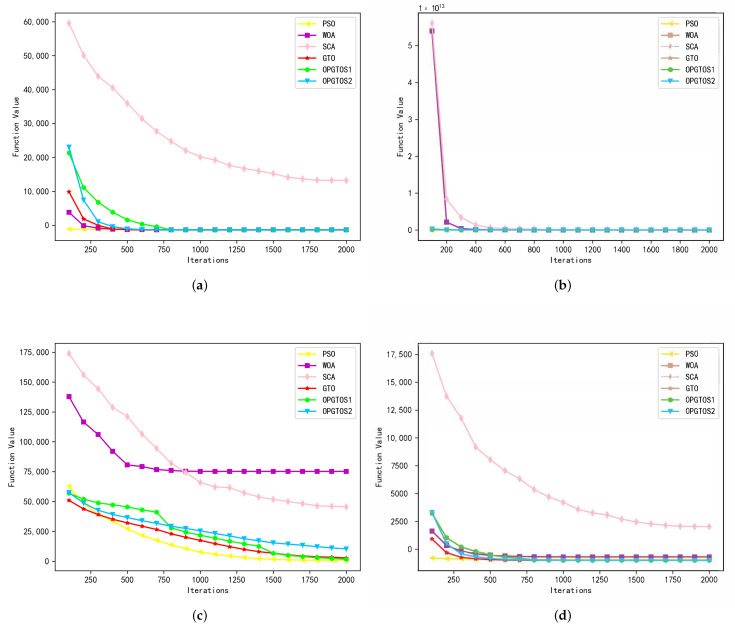
Simulation results of unimodal functions in 30 dimensions. (**a**) F1. (**b**) F3. (**c**) F4. (**d**) F5.

**Figure 7:**
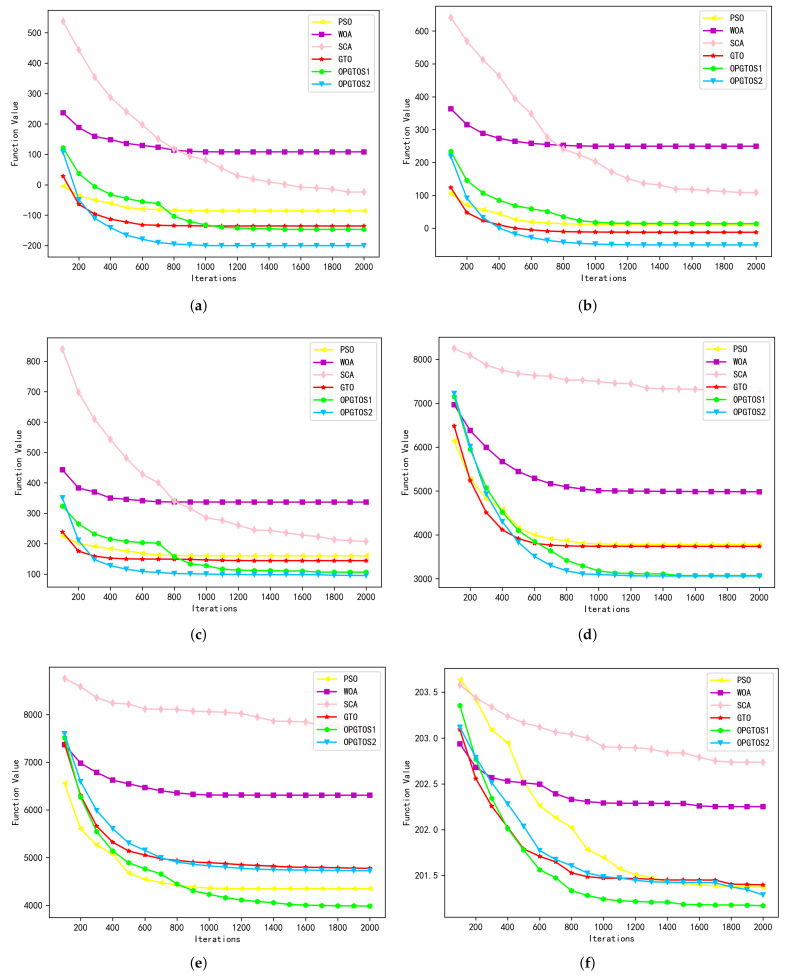
Simulation results of basic multimodal functions in 30 dimensions. (**a**) F11. (**b**) F12. (**c**) F13. (**d**) F14. (**e**) F15. (**f**) F16.

**Figure 8:**
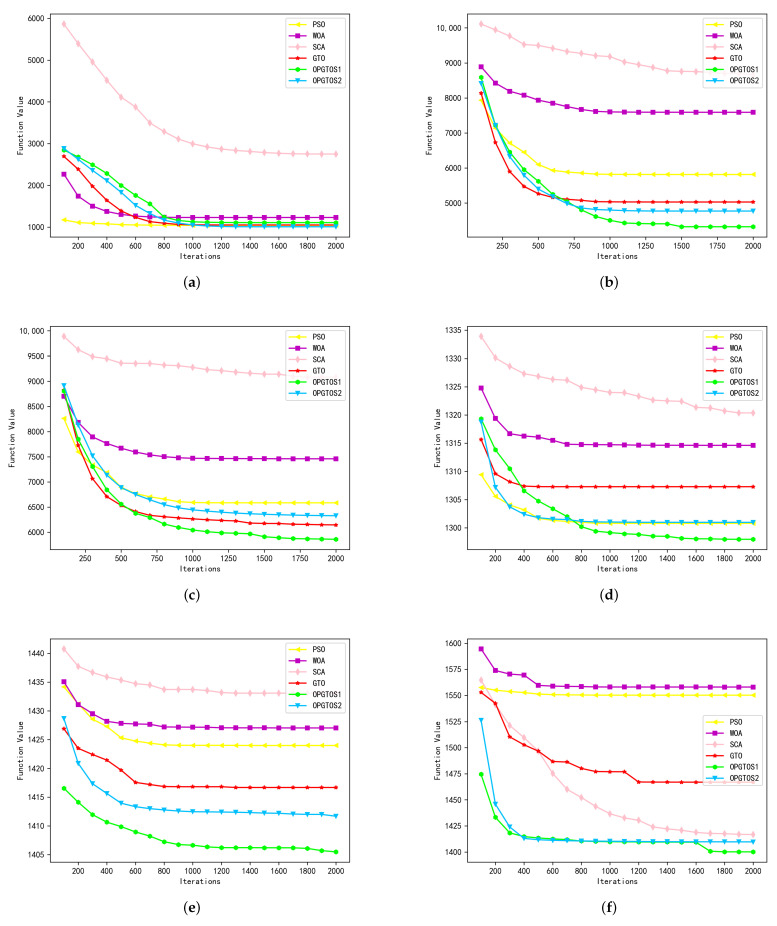

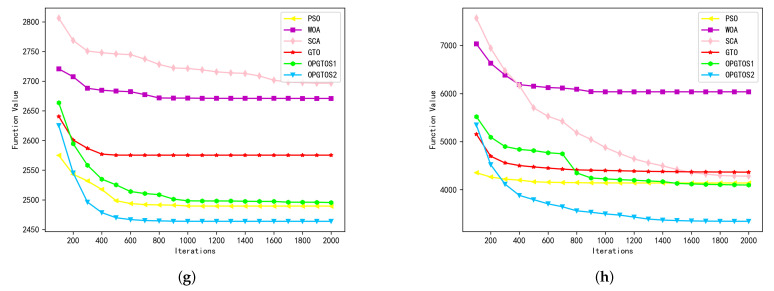
Simulation results of composition functions in 30 dimensions. (**a**) F21. (**b**) F22. (**c**) F23. (**d**) F24. (**e**) F25. (**f**) F26. (**g**) F27. (**h**) F28.

As shown in Table 3, all algorithms are tested in 30 dimensions. Among the 28 tested functions, OPGTOS1 achieves 10 better solutions and 2 identical solutions, while OPGTOS2 achieves 8 better solutions and 1 identical solution compared to other algorithms. Compared with the GTO algorithm, OPGTOS1 has achieved 20 better solutions and 2 identical solutions, and OPGTOS2 has achieved 21 better solutions and 1 identical solution. In order to analyze the iterative process of different algorithms more clearly, the experiment records the data of the optimal solution every 100 iterations in detail in 30 dimensions and draws it as Figure 6, Figure 7 and Figure 8. In these figures, the horizontal axis is the number of iterations of the algorithm and the vertical axis is the optimal solution for the corresponding number of iterations.

According to Table 3, and Figure 6, the experimental results show that multi-group merge communication strategy is the best choice for finding the optimal value of a unimodal function. The test results of functions F1, F3, F4 and F5 show that the optimal value searched by OPGTOS1 is much smaller than the traditional optimization algorithm and the original GTO algorithm. Especially in function F4, the convergence speed of OPGTOS1 increases significantly around 700 generations and 1400 iterations, because the algorithm performs group merging every 700 generations. Experiments show that the merged group not only inherits the advantages of the original group, but also achieves effective mutation.

OPGTOS1 and OPGTO2 perform extremely well when solving multimodal functions. For functions F11 and F12, OPGTOS2 not only has the fastest convergence speed and convergence stability, but also achieves the optimal value far smaller than other algorithms, which indicates that the algorithm has strong global exploration ability. In the mutation and update phase of strategy 2, many individuals will learn from the optimal individual, which is beneficial for the algorithm to quickly approach the optimal solution. In the test functions F13 and F14, compared with other algorithms, both OPGTOS1 and OPGTO2 achieve better optimal solutions. For functions F15 and F16, OPGTOS1 has the fastest convergence speed and the most suitable solution. It can be seen from Figure 7 that strategy 1 converges quickly when merging groups, which indicates that the merging operation brings more possibilities for the algorithm to find the optimal solution.

F21–F28 belong to more complex combinatorial functions, and in the experimental results of these functions, OPGTOS1 and OPGTOS2 have achieved all the leading positions. In functions F22–F26, the solution found by OPGTOS1 is much smaller than other algorithms. It can be seen from the Figure 8 that OPGTOS1 will have some rapid declines on the convergence curves of these functions, which indicates that the Gauss-Cauchy mutation strategy has achieved good results, and the algorithm has found a better solution. Among the functions F27 and F28, OPGTOS2 has the fastest convergence speed, and the obtained optimal solution is also the smallest.

In Table 4, all algorithms are tested in 50 dimensions. Compared to other algorithms, OPGTOS1 achieves 12 better solutions and 1 identical solution, and OPGTOS2 achieves 7 better solutions. Compared with the GTO algorithm, OPGTOS1 achieved 20 better solutions and 1 identical solution, and OPGTOS2 achieved 18 better solutions. OPGTOS1 shows far better performance than other optimization algorithms on single-modal functions and complex combination functions, and OPGTOS2 has better performance on multi-modal functions.

We take the average solution time of each algorithm for all functions of CEC2013 as the resource consumption of the algorithm. In Table 5, PSO, WOA, and SCA have simple exploration and development mechanisms, so the average solution time of the algorithm is shorter, but the optimal solution is poor. GTO has more complex operations, which leads to longer solution times. Compared with GTO, our proposed OPGTOS1 and OPGTOS2 have more mutation and update mechanisms. Although the resource consumption of the algorithm increases, a more suitable optimal solution is obtained.

### 4.2. Simulation Results of OPGTO in 3D Localization

**Figure 9:**
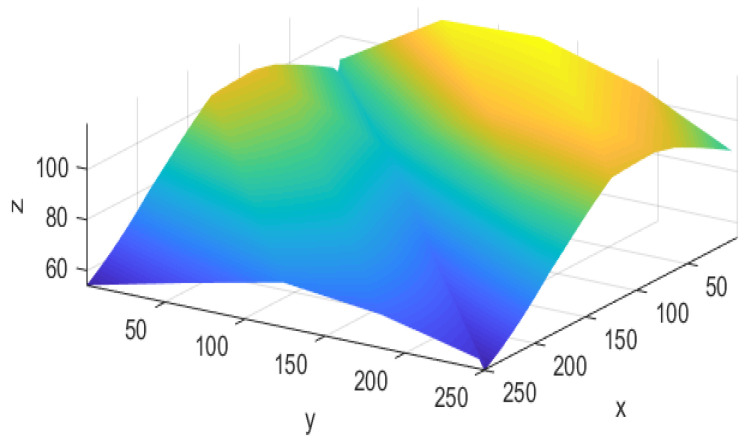
3D terrain of Bijia Mountain.

In this section, OPGTO is applied to optimize node localization in a piece of actual 3D terrain. The terrain chosen for the experiment is Bijia Mountain in Qingdao. The terrain of this mountain is complex and rugged with peaks, ravines and valleys. It is very suitable for simulating node localization in real terrain and can also test various performances of the algorithm more efficiently. The 3D terrain used in this paper is first extracted using Google Maps and then drawn using Matlab, as shown in Figure 7. When a node in WSN communicates with other nodes in the area, the node may not be able to communicate properly. The reason may be that the communication distance exceeds the transmission distance of the node or the signal will be blocked by objects on the mountain. Therefore, we need to first detect whether the nodes can communicate normally. In three-dimensional space, the communication range of sensor nodes resembles a sphere. If the communication distance between two nodes is greater than the transmission distance of the nodes, it indicates that the communication ranges of the two nodes do not cover each other. As a result, the nodes cannot communicate properly. Then using the LOS method, we can effectively determine whether there are obstacles between nodes. Figure 9 shows the result of this process, *x*, *y* and *z* represent the three-dimensional space coordinates of a point in the terrain.

**Figure 10:**
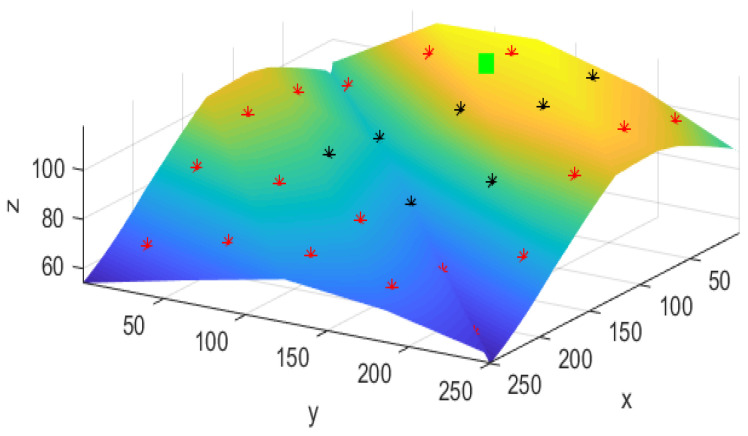
Communication status between nodes.

In Figure 10, 25 anchor nodes and a randomly generated unknown node are included. The green square represents an unknown node, the black marker represents the anchor node that the unknown node can communicate with normally, and the red marker represents the anchor node that the unknown node cannot reach. In order to represent the localization error, the fitness function calculation formula is proposed in this paper as follows:

(27) $$\;f\left(x,y\right)=min\left(\sqrt{\frac{error}{N-1}}\right)$$

In Equation (27), *error* is calculated by Equation (24) and *N* represents the number of anchor nodes that the unknown node can communicate normally. The experiment sets 25 anchor nodes, which are uniformly distributed in a 3D terrain of size 100 × 250 × 250. To comprehensively test the performance of OPGTO in TDOA, we randomly sprinkle unknown nodes into the terrain and locate them. At the same time, we change the signal transmission distance of the node and detect the change of localization error under different transmission distances. Signal transmission distance is a crucial factor for normal communication between sensor nodes. If the transmission distance of the signal is too long, the node will consume more energy to transmit information to anchor nodes. In addition, for anchor nodes that have established communication connections. Too long transmission distance will inevitably increase the time error of signal arrival, which will have an impact on the final node location accuracy. If the transmission distance is too short, the node cannot communicate with any anchor node and send its own location information.

**Figure 11:**
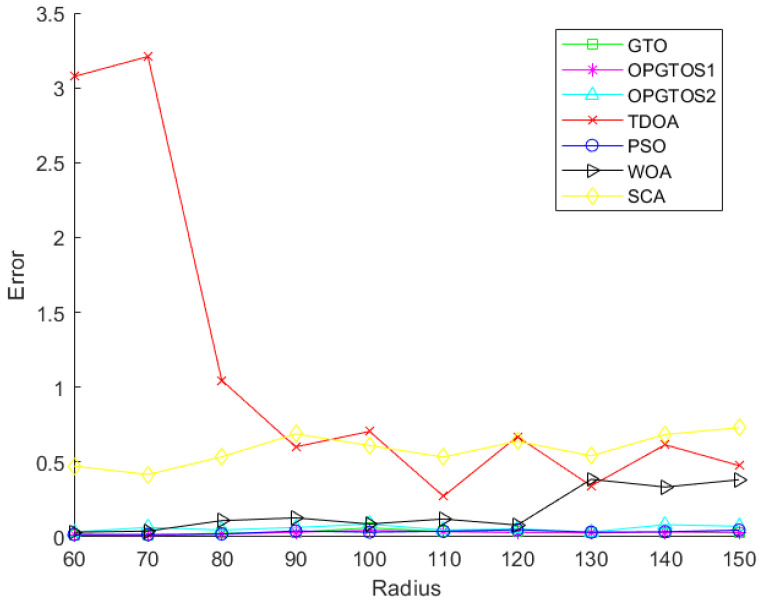
The localization error of TDOA and the optimization algorithms.

**Figure 12:**
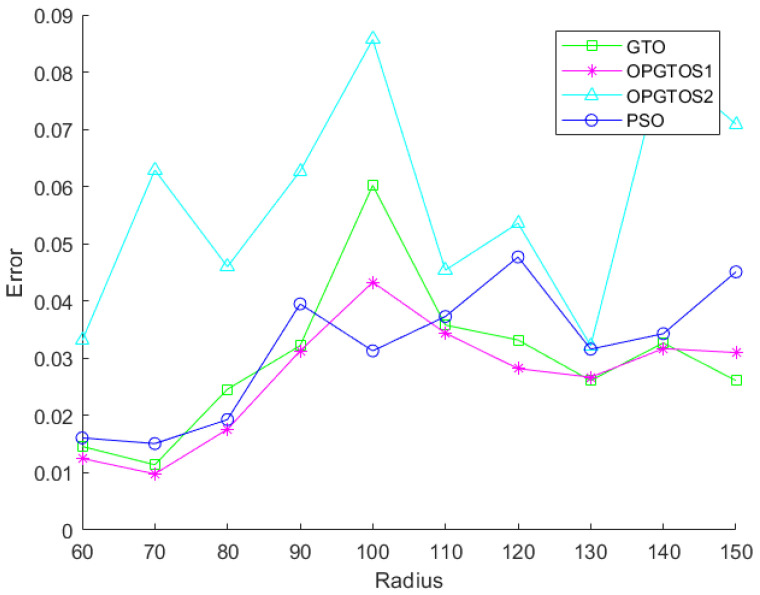
The localization error of PSO, GTO, OPGTOS1 and OPGTOS2.

In Figure 11, the x-axis represents the detection radius of the node and the y-axis represents the corresponding localization error. Figure 11 shows the errors of TDOA, PSO, WOA, SCA, GTO, OPGTOS1 and OPGTOS2 under different detection radius, and we can see that the error rate of the optimization algorithms is much smaller than TDOA. Figure 12 shows the positioning errors of PSO, GTO, OPGTOS1 and OPGTOS2. When the detection radius is between 60 m and 120 m, the localization error rate of OPGTOS1 is smaller than other algorithms. In addition, its variation range is smaller than other algorithms, which indicates that the stability of the algorithm is better.

## 5. Discussion

The main innovations of the OPGTO algorithm proposed in this paper are opposition-based learning and multi-group communication strategies. From Table 2, Table 3 and Table 4, it can be found that OPGTO leads GTO in solving multimodal and combinatorial functions. This illustrates that our proposed strategy improves the global exploration ability of the algorithm. Compared with PPSO [34], PWOA [40], PCSCA [9], we innovatively combine the strategy of opposition-based learning, which has been widely used to extend reinforcement learning and neural networks. It helps us to achieve better optimal solution and faster convergence curve in CEC2013, which further reduces the error in 3D localization of WSN. However, the running speed of OPGTO is limited to a certain extent due to more complicated operations and strategies.

There are many other improvement strategies for GTO as well. For example, two major improvements to MIGTO [43] are Explorative Gorilla with Adaptive Mutation Mechanism (EGAMM) and Gorilla Memory Saving Technology. Different from MIGTO, we propose two communication strategies between groups based on the idea of parallel. Communication strategies play a huge role in the exploration and development of particle populations, especially when algorithm faces complex optimization problems.

## 6. Conclusions and Feature Works

In this paper, we propose a GTO algorithm based on opposition-based learning and parallel strategy. OPGTO has demonstrated extraordinary ability in terms of convergence speed and global exploration. We conduct extensive experiments on the CEC2013 benchmark functions in 10, 30, and 50 dimensions, and the simulation results demonstrate OPGTO’s particularly robust exploration capabilities. Compared with the existing traditional optimization algorithms PSO, WOA, SCA and the original GTO algorithm, OPGTO has more appropriate global optimal solutions and faster convergence. Especially in solving more complex multimodal functions and combinatorial functions, OPGTO has shown far superior performance than other algorithms. Finally, we apply the OPGTO algorithm to the node localization of WSN in 3D real terrain and obtain smaller localization errors than the TDOA method, which further verifies the ability of the new algorithm to solve optimization problems. However, OPGTO has more operations in the way of complex mutations and updates. This results in the algorithm requiring more computing power consumption and resource occupation. In the future, we will consider using compact [9,27,44] strategies to reduce the time complexity of the algorithm. The compact method is a population-free method, which is essentially a probabilistic model based on the Estimated Distribution Algorithm (EDA). During the execution of the algorithm, the distribution model will replace the population to perform related operations, and the continuous search of the exploration space is realized by updating the distribution model. Furthermore, we will combine some promising technologies [44,45,46,47] to make more improvements on the existing algorithms and expand more application fields [48,49,50].

## Figures and Tables

**Table 1:** Parameter settings for each algorithm.

Algorithm	Parameter
PSO	c = 2.0, w = 0.9, pop_size = 40, iterations = 2000
WOA	a = 2.0, b = 1, pop_size = 40, iterations = 2000
SCA	a = 2.0, pop_size = 40, iterations = 2000
GTO	p = 0.03, β = 3, W = 0.8, pop_size = 40, iterations = 2000
OPGTOS1	p = 0.03, β = 3, W = 0.8, groups = 4, pop_size = 40, iterations = 2000
OPGTOS2	p = 0.03, β = 3, W = 0.8, groups = 4, pop_size = 40, iterations = 2000

**Table 2:** Simulation results of CEC2013 functions in 10 dimensions.

Functions	PSO	WOA	SCA	GTO	OPGTOS1	OPGTOS2
F1	**−1.4000 × 10${}^{3}$**	**−1.4000 × 10${}^{3}$**	−8.3476 × 10${}^{2}$	**−1.4000 × 10${}^{3}$**	**−1.4000 × 10${}^{3}$**	**−1.4000 × 10${}^{3}$**
F2	4.8719 × 10${}^{4}$	2.8500 × 10${}^{6}$	3.4431 × 10${}^{6}$	4.9012 × 10${}^{3}$	**4.2691 × 10${}^{3}$**	1.8311 × 10${}^{4}$
F3	3.9529 × 10${}^{7}$	1.4331 × 10${}^{9}$	7.1982 × 10${}^{8}$	**2.4489 × 10${}^{7}$**	2.5205 × 10${}^{7}$	7.6459 × 10${}^{7}$
F4	−1.0871 × 10${}^{3}$	2.5367 × 10${}^{4}$	3.9081 × 10${}^{3}$	−1.0911 × 10${}^{3}$	**−1.0989 × 10${}^{3}$**	−1.0031 × 10${}^{3}$
F5	**−1.0000 × 10${}^{3}$**	−9.7888 × 10${}^{2}$	−8.6577 × 10${}^{2}$	**−1.0000 × 10${}^{3}$**	**−1.0000 × 10${}^{3}$**	**−1.0000 × 10${}^{3}$**
F6	−8.9274 × 10${}^{2}$	−8.6231 × 10${}^{2}$	−8.4421 × 10${}^{2}$	−8.9150 × 10${}^{2}$	**−8.9772 × 10${}^{2}$**	−8.9166 × 10${}^{2}$
F7	−7.5654 × 10${}^{2}$	−7.1223 × 10${}^{2}$	−7.5606 × 10${}^{2}$	−7.5235 × 10${}^{2}$	−7.6200 × 10${}^{2}$	**−7.6313 × 10${}^{2}$**
F8	−6.7968 × 10${}^{2}$	−6.7967 × 10${}^{2}$	−6.7964 × 10${}^{2}$	−6.7965 × 10${}^{2}$	**−6.7968 × 10${}^{2}$**	−6.7967 × 10${}^{2}$
F9	−5.9409 × 10${}^{2}$	−5.9271 × 10${}^{2}$	−5.9211 × 10${}^{2}$	−5.9407 × 10${}^{2}$	**−5.9508 × 10${}^{2}$**	−5.9489 × 10${}^{2}$
F10	−4.9850 × 10${}^{2}$	−4.8844 × 10${}^{2}$	−4.1692 × 10${}^{2}$	−4.9822 × 10${}^{2}$	**−4.9932 × 10${}^{2}$**	−4.9882 × 10${}^{2}$
F11	−3.7289 × 10${}^{2}$	−3.2896 × 10${}^{2}$	−3.4374 × 10${}^{2}$	−3.7712 × 10${}^{2}$	−3.7751 × 10${}^{2}$	**−3.8312 × 10${}^{2}$**
F12	−2.6204 × 10${}^{2}$	−2.1853 × 10${}^{2}$	−2.4195 × 10${}^{2}$	−2.6748 × 10${}^{2}$	−2.6781 × 10${}^{2}$	**−2.6841 × 10${}^{2}$**
F13	−1.5345 × 10${}^{2}$	−1.3115 × 10${}^{2}$	−1.4399 × 10${}^{2}$	−1.5926 × 10${}^{2}$	−1.6091 × 10${}^{2}$	**−1.6431 × 10${}^{2}$**
F14	6.4271 × 10${}^{2}$	9.1978 × 10${}^{2}$	1.1730 × 10${}^{3}$	4.9112 × 10${}^{2}$	**3.7030 × 10${}^{2}$**	4.3141 × 10${}^{2}$
F15	8.1545 × 10${}^{2}$	1.2740 × 10${}^{3}$	1.5704 × 10${}^{3}$	8.7427 × 10${}^{2}$	**6.3787 × 10${}^{2}$**	7.0925 × 10${}^{2}$
F16	2.0057 × 10${}^{2}$	2.0081 × 10${}^{2}$	2.0121 × 10${}^{2}$	2.0046 × 10${}^{2}$	**2.0041 × 10${}^{2}$**	2.0043 × 10${}^{2}$
F17	**3.2301 × 10${}^{2}$**	3.7425 × 10${}^{2}$	3.6092 × 10${}^{2}$	3.3686 × 10${}^{2}$	3.3341 × 10${}^{2}$	3.3634 × 10${}^{2}$
F18	**4.2450 × 10${}^{2}$**	4.7709 × 10${}^{2}$	4.6342 × 10${}^{2}$	4.4208 × 10${}^{2}$	4.4060 × 10${}^{2}$	4.3527 × 10${}^{2}$
F19	**5.0095 × 10${}^{2}$**	5.0670 × 10${}^{2}$	5.1151 × 10${}^{2}$	5.0180 × 10${}^{2}$	5.0190 × 10${}^{2}$	**5.0159 × 10${}^{2}$**
F20	6.0319 × 10${}^{2}$	6.0396 × 10${}^{2}$	6.0341 × 10${}^{2}$	6.0332 × 10${}^{2}$	**6.0318 × 10${}^{2}$**	6.0319 × 10${}^{2}$
F21	1.1002 × 10${}^{3}$	1.0606 × 10${}^{3}$	1.1131 × 10${}^{3}$	1.1002 × 10${}^{3}$	**1.0902 × 10${}^{3}$**	1.1002 × 10${}^{3}$
F22	1.9130 × 10${}^{3}$	2.2000 × 10${}^{3}$	2.3646 × 10${}^{3}$	1.5850 × 10${}^{3}$	**1.3574 × 10${}^{3}$**	1.5500 × 10${}^{3}$
F23	2.1078 × 10${}^{3}$	2.5235 × 10${}^{3}$	2.6434 × 10${}^{3}$	2.2005 × 10${}^{3}$	**1.8884 × 10${}^{3}$**	1.9143 × 10${}^{3}$
F24	1.2204 × 10${}^{3}$	1.2212 × 10${}^{3}$	1.2259 × 10${}^{3}$	1.1974 × 10${}^{3}$	**1.1723 × 10${}^{3}$**	1.1754 × 10${}^{3}$
F25	1.3194 × 10${}^{3}$	1.3247 × 10${}^{3}$	1.3192 × 10${}^{3}$	1.2868 × 10${}^{3}$	1.2800 × 10${}^{3}$	**1.2702 × 10${}^{3}$**
F26	1.4499 × 10${}^{3}$	1.3972 × 10${}^{3}$	1.3988 × 10${}^{3}$	1.3964 × 10${}^{3}$	1.3644 × 10${}^{3}$	**1.3616 × 10${}^{3}$**
F27	1.7437 × 10${}^{3}$	1.8893 × 10${}^{3}$	1.9051 × 10${}^{3}$	1.8341 × 10${}^{3}$	**1.7000 × 10${}^{3}$**	1.7078 × 10${}^{3}$
F28	2.0522 × 10${}^{3}$	2.1678 × 10${}^{3}$	2.0683 × 10${}^{3}$	1.9700 × 10${}^{3}$	**1.9667 × 10${}^{3}$**	2.0163 × 10${}^{3}$

Bold indicates the optimal solution to the test function.

**Table 3:** Simulation results of CEC2013 functions in 30 dimensions.

Functions	PSO	WOA	SCA	GTO	OPGTOS1	OPGTOS2
F1	−1.2584 × 10${}^{3}$	−1.3351 × 10${}^{3}$	1.3195 × 10${}^{4}$	**−1.4000 × 10${}^{3}$**	**−1.4000 × 10${}^{3}$**	**−1.4000 × 10${}^{3}$**
F2	2.2255 × 10${}^{6}$	7.3459 × 10${}^{7}$	1.8283 × 10${}^{8}$	**1.7558 × 10${}^{6}$**	1.9812 × 10${}^{6}$	4.0057 × 10${}^{6}$
F3	7.3140 × 10${}^{9}$	3.1777 × 10${}^{10}$	5.1810 × 10${}^{10}$	2.1691 × 10${}^{9}$	**1.3189 × 10${}^{9}$**	1.9336 × 10${}^{9}$
F4	7.6646 × 10${}^{2}$	7.5215 × 10${}^{4}$	4.5524 × 10${}^{4}$	2.7143 × 10${}^{3}$	**1.5924 × 10${}^{3}$**	1.0304 × 10${}^{4}$
F5	−9.3156 × 10${}^{2}$	−6.9280 × 10${}^{2}$	2.0185 × 10${}^{3}$	**−1.0000 × 10${}^{3}$**	**−1.0000 × 10${}^{3}$**	−9.9999 × 10${}^{2}$
F6	−8.3101 × 10${}^{2}$	−7.2677 × 10${}^{2}$	1.2517 × 10${}^{2}$	−8.3725 × 10${}^{2}$	**−8.3792 × 10${}^{2}$**	−8.3341 × 10${}^{2}$
F7	−6.6617 × 10${}^{2}$	−3.6581 × 10${}^{2}$	−5.8299 × 10${}^{2}$	−6.5157 × 10${}^{2}$	−6.7202 × 10${}^{2}$	**−6.7829 × 10${}^{2}$**
F8	**−6.7905 × 10${}^{2}$**	−6.7902 × 10${}^{2}$	−6.7900 × 10${}^{2}$	−6.7903 × 10${}^{2}$	−6.7904 × 10${}^{2}$	−6.7901 × 10${}^{2}$
F9	**−5.6993 × 10${}^{2}$**	−5.6310 × 10${}^{2}$	−5.5912 × 10${}^{2}$	−5.6529 × 10${}^{2}$	−5.6846 × 10${}^{2}$	−5.6716 × 10${}^{2}$
F10	−4.5624 × 10${}^{2}$	−2.6043 × 10${}^{2}$	1.4309 × 10${}^{3}$	**−4.9968 × 10${}^{2}$**	−4.9937 × 10${}^{2}$	−4.9778 × 10${}^{2}$
F11	−8.5753 × 10	1.0837 × 10${}^{2}$	−2.3930 × 10	−1.3550 × 10${}^{2}$	−1.4663 × 10${}^{2}$	**−2.0006 × 10${}^{2}$**
F12	1.1691 × 10	2.4957 × 10${}^{2}$	1.0852 × 10${}^{2}$	−1.2259 × 10	1.3963 × 10	**−5.0813 × 10**
F13	1.5989 × 10${}^{2}$	3.3666 × 10${}^{2}$	2.0759 × 10${}^{2}$	1.4414 × 10${}^{2}$	1.0630 × 10${}^{2}$	**9.5097 × 10**
F14	3.7839 × 10${}^{3}$	4.9830 × 10${}^{3}$	7.2659 × 10${}^{3}$	3.7392 × 10${}^{3}$	3.0732 × 10${}^{3}$	**3.0626 × 10${}^{3}$**
F15	4.3491 × 10${}^{3}$	6.3085 × 10${}^{3}$	7.7583 × 10${}^{3}$	4.7744 × 10${}^{3}$	**3.9834 × 10${}^{3}$**	4.7250 × 10${}^{3}$
F16	2.0137 × 10${}^{2}$	2.0225 × 10${}^{2}$	2.0274 × 10${}^{2}$	2.0140 × 10${}^{2}$	**2.0117 × 10${}^{2}$**	2.0129 × 10${}^{2}$
F17	**5.0260 × 10${}^{2}$**	9.6147 × 10${}^{2}$	8.5302 × 10${}^{2}$	6.8331 × 10${}^{2}$	6.3125 × 10${}^{2}$	6.1835 × 10${}^{2}$
F18	**5.8747 × 10${}^{2}$**	1.0157 × 10${}^{3}$	9.2840 × 10${}^{2}$	7.5694 × 10${}^{2}$	7.9862 × 10${}^{2}$	7.0279 × 10${}^{2}$
F19	**5.0917 × 10${}^{2}$**	5.8332 × 10${}^{2}$	8.8438 × 10${}^{3}$	5.5840 × 10${}^{2}$	5.2631 × 10${}^{2}$	5.2416 × 10${}^{2}$
F20	6.1458 × 10${}^{2}$	6.1490 × 10${}^{2}$	6.1419 × 10${}^{2}$	**6.1333 × 10${}^{2}$**	6.1337 × 10${}^{2}$	6.1334 × 10${}^{2}$
F21	1.0419 × 10${}^{3}$	1.2307 × 10${}^{3}$	2.7498 × 10${}^{3}$	1.0518 × 10${}^{3}$	1.1077 × 10${}^{3}$	**1.0087 × 10${}^{3}$**
F22	5.8150 × 10${}^{3}$	7.5899 × 10${}^{3}$	8.6594 × 10${}^{3}$	5.0295 × 10${}^{3}$	**4.3238 × 10${}^{3}$**	4.7720 × 10${}^{3}$
F23	6.5847 × 10${}^{3}$	7.4593 × 10${}^{3}$	9.0820 × 10${}^{3}$	6.1455 × 10${}^{3}$	**5.8594 × 10${}^{3}$**	6.3281 × 10${}^{3}$
F24	1.3008 × 10${}^{3}$	1.3146 × 10${}^{3}$	1.3204 × 10${}^{3}$	1.3073 × 10${}^{3}$	**1.2980 × 10${}^{3}$**	1.3010 × 10${}^{3}$
F25	1.4240 × 10${}^{3}$	1.4270 × 10${}^{3}$	1.4331 × 10${}^{3}$	1.4167 × 10${}^{3}$	**1.4055 × 10${}^{3}$**	1.4117 × 10${}^{3}$
F26	1.5502 × 10${}^{3}$	1.5580 × 10${}^{3}$	1.4167 × 10${}^{3}$	1.4669 × 10${}^{3}$	**1.4001 × 10${}^{3}$**	1.4097 × 10${}^{3}$
F27	2.4895 × 10${}^{3}$	2.6709 × 10${}^{3}$	2.6966 × 10${}^{3}$	2.5753 × 10${}^{3}$	2.4954 × 10${}^{3}$	**2.4638 × 10${}^{3}$**
F28	4.1396 × 10${}^{3}$	6.0367 × 10${}^{3}$	4.2822 × 10${}^{3}$	4.3647 × 10${}^{3}$	4.0969 × 10${}^{3}$	**3.3420 × 10${}^{3}$**

Bold indicates the optimal solution to the test function.

**Table 4:** Simulation results of CEC2013 functions in 50 dimensions.

Functions	PSO	WOA	SCA	GTO	OPGTOS1	OPGTOS2
F1	−1.2309 × 10${}^{3}$	−7.9109 × 10${}^{2}$	3.2256 × 10${}^{4}$	**−1.4000 × 10${}^{3}$**	**−1.4000 × 10${}^{3}$**	−1.4000 × 10${}^{3}$
F2	8.7200 × 10${}^{6}$	1.2286 × 10${}^{8}$	6.0050 × 10${}^{8}$	6.1892 × 10${}^{6}$	**5.6450 × 10${}^{6}$**	1.3470 × 10${}^{7}$
F3	8.1706 × 10${}^{9}$	5.5928 × 10${}^{10}$	1.3181 × 10${}^{11}$	6.7839 × 10${}^{9}$	**4.8846 × 10${}^{9}$**	7.3252 × 10${}^{9}$
F4	6.9780 × 10${}^{3}$	7.6695 × 10${}^{4}$	7.6340 × 10${}^{4}$	1.1636 × 10${}^{4}$	**1.0673 × 10${}^{4}$**	2.1880 × 10${}^{4}$
F5	−9.5832 × 10${}^{2}$	−3.1282 × 10${}^{2}$	3.6436 × 10${}^{3}$	**−1.0000 × 10${}^{3}$**	−1.0000 × 10${}^{3}$	−9.9999 × 10${}^{2}$
F6	−7.8109 × 10${}^{2}$	−5.4184 × 10${}^{2}$	1.7422 × 10${}^{3}$	**−8.0498 × 10${}^{2}$**	−8.0446 × 10${}^{2}$	−7.9179 × 10${}^{2}$
F7	−6.7300 × 10${}^{2}$	4.9938 × 10${}^{2}$	−5.4663 × 10${}^{2}$	−6.2500 × 10${}^{2}$	−6.7626 × 10${}^{2}$	**−6.7978 × 10${}^{2}$**
F8	**−6.7885 × 10${}^{2}$**	−6.7883 × 10${}^{2}$	−6.7880 × 10${}^{2}$	−6.7883 × 10${}^{2}$	−6.7883 × 10${}^{2}$	−6.7884 × 10${}^{2}$
F9	**−5.4053 × 10${}^{2}$**	−5.2915 × 10${}^{2}$	−5.2499 × 10${}^{2}$	−5.3580 × 10${}^{2}$	−5.3903 × 10${}^{2}$	−5.3883 × 10${}^{2}$
F10	−3.9532 × 10${}^{2}$	1.2906 × 10${}^{2}$	3.9252 × 10${}^{3}$	**−4.9399 × 10${}^{2}$**	−4.9172 × 10${}^{2}$	−4.6296 × 10${}^{2}$
F11	1.0823 × 10${}^{2}$	4.6861 × 10${}^{2}$	3.7045 × 10${}^{2}$	1.0867 × 10${}^{2}$	4.3230 × 10	**8.6476**
F12	2.7200 × 10${}^{2}$	6.6069 × 10${}^{2}$	5.1083 × 10${}^{2}$	2.7110 × 10${}^{2}$	3.0672 × 10${}^{2}$	**1.9867 × 10${}^{2}$**
F13	4.6241 × 10${}^{2}$	8.5380 × 10${}^{2}$	5.9303 × 10${}^{2}$	5.6585 × 10${}^{2}$	4.3875 × 10${}^{2}$	**4.0373 × 10${}^{2}$**
F14	7.2073 × 10${}^{3}$	1.0615 × 10${}^{4}$	1.3649 × 10${}^{4}$	6.5409 × 10${}^{3}$	5.7605 × 10${}^{3}$	**5.6061 × 10${}^{3}$**
F15	9.0707 × 10${}^{3}$	1.1938 × 10${}^{4}$	1.4869 × 10${}^{4}$	9.5541 × 10${}^{3}$	**8.1922 × 10${}^{3}$**	9.6221 × 10${}^{3}$
F16	2.0224 × 10${}^{2}$	2.0272 × 10${}^{2}$	2.0378 × 10${}^{2}$	2.0206 × 10${}^{2}$	2.0206 × 10${}^{2}$	**2.0203 × 10${}^{2}$**
F17	**7.9120 × 10${}^{2}$**	1.4946 × 10${}^{3}$	1.3869 × 10${}^{3}$	1.0208 × 10${}^{3}$	1.0331 × 10${}^{3}$	9.9202 × 10${}^{2}$
F18	**8.2142 × 10${}^{2}$**	1.5835 × 10${}^{3}$	1.4754 × 10${}^{3}$	1.1978 × 10${}^{3}$	1.1768 × 10${}^{3}$	1.0739 × 10${}^{3}$
F19	**5.3004 × 10${}^{2}$**	8.0833 × 10${}^{2}$	6.4222 × 10${}^{4}$	6.5196 × 10${}^{2}$	5.7426 × 10${}^{2}$	5.9644 × 10${}^{2}$
F20	6.2389 × 10${}^{2}$	6.2472 × 10${}^{2}$	6.2420 × 10${}^{2}$	6.2327 × 10${}^{2}$	**6.2303 × 10${}^{2}$**	6.2392 × 10${}^{2}$
F21	1.6093 × 10${}^{3}$	2.5666 × 10${}^{3}$	4.7081 × 10${}^{3}$	1.6079 × 10${}^{3}$	**1.5936 × 10${}^{3}$**	1.6222 × 10${}^{3}$
F22	1.1971 × 10${}^{4}$	1.3664 × 10${}^{4}$	1.5815 × 10${}^{4}$	9.3579 × 10${}^{3}$	**7.6946 × 10${}^{3}$**	7.9538 × 10${}^{3}$
F23	1.2290 × 10${}^{4}$	1.4687 × 10${}^{4}$	1.6388 × 10${}^{4}$	1.2314 × 10${}^{4}$	**1.0373 × 10${}^{4}$**	1.1825 × 10${}^{4}$
F24	1.3894 × 10${}^{3}$	1.4260 × 10${}^{3}$	1.4317 × 10${}^{3}$	1.3966 × 10${}^{3}$	**1.3824 × 10${}^{3}$**	1.3883 × 10${}^{3}$
F25	1.5269 × 10${}^{3}$	1.5327 × 10${}^{3}$	1.5530 × 10${}^{3}$	1.5279 × 10${}^{3}$	**1.4976 × 10${}^{3}$**	1.5109 × 10${}^{3}$
F26	1.6460 × 10${}^{3}$	1.6469 × 10${}^{3}$	1.6219 × 10${}^{3}$	1.5892 × 10${}^{3}$	**1.5046 × 10${}^{3}$**	1.5335 × 10${}^{3}$
F27	3.3618 × 10${}^{3}$	3.6134 × 10${}^{3}$	3.6892 × 10${}^{3}$	3.3915 × 10${}^{3}$	**3.3555 × 10${}^{3}$**	3.3588 × 10${}^{3}$
F28	4.2321 × 10${}^{3}$	9.9957 × 10${}^{3}$	7.0825 × 10${}^{3}$	4.8026 × 10${}^{3}$	4.7862 × 10${}^{3}$	**3.7541 × 10${}^{3}$**

Bold indicates the optimal solution to the test function.

**Table 5:** Average solution time on CEC2013 in 30 dimensions.

Functions	PSO	WOA	SCA	GTO	OPGTOS1	OPGTOS2
Runtime(s)	44.015153	39.547344	53.934907	112.934504	163.106233	171.35796

## Data Availability

Not applicable.
